# Diagnostic value of radionuclide in bone metastasis after breast cancer surgery: A protocol of systematic review

**DOI:** 10.1097/MD.0000000000021585

**Published:** 2020-08-14

**Authors:** Qi-xin Lian, Wei Zhao, Gang Li, Lian-jin Jin, Hao-jie Nie

**Affiliations:** a Department of Tumor Surgery, The First Affiliated Hospital of Jiamusi University, Jiamusi; b Department of Anatomy, Mudanjiang Medical University; c First Ward of Orthopedics Department; d Department of Anesthesiology, The Affiliated Hongqi Hospital of Mudanjiang Medical University, Mudanjiang; e Department of Isotope, The First Affiliated Hospital of Heilongjiang University of Traditional Chinese Medicine, Harbin, China.

**Keywords:** breast cancer, bone metastasis, diagnosis, radionuclide

## Abstract

**Background:**

The objective of this study is to evaluate the accuracy of radionuclide in diagnosis of bone metastasis (BM) after breast cancer surgery (BCS).

**Methods:**

The electronic databases (Cochrane Library, MEDLINE, EMBASE, Web of Science, CBM, and CNKI) will be systematically and comprehensively searched until June 1, 2020 for eligible studies that reported the diagnosis of radionuclide in BM after BCS. In addition, we will also identify grey literatures, such as conference abstracts, and reference lists of included studies. All process of study identification, data extraction, and study methodological quality evaluation will be performed by 2 independent authors. All divergences will be settled by a third author through discussion. All data analysis will be carried out by RevMan 5.3 software (London, UK).

**Results:**

This study will scrutinize the most recent evidence of radionuclide in detection of BM after BCS.

**Conclusion:**

This study may provide evidence of accuracy of radionuclide in diagnosis of BM following BCS.

**Study registration number:**

PROSPERO CRD42020187646.

## 1 Introduction

Breast cancer (BC) is one of the most frequently diagnosed gynecological cancers,^[1,2]^ which is the second leading cause of cancer mortality.^[3,4]^ It is reported that about 90% to 95% patients are diagnosed at early stage and 20% to 30% of them have metastatic.^[5]^ Bone metastatic (BM) is the most frequent metastases in patients with BC,^[6,7]^ with >75% of stage IV BC develop to BM, which is incurable for these patients.^[8,9]^ Thus, it is very to treat BC at early stage. Although surgery is a mostly utilized management for patients with BC, there are still some patients suffering from BM after surgery.^[10]^ It is very important to detect BM in patients after breast cancer surgery (BCS).

Radionuclide is reported to diagnose BM after BCS.^[11]^ Despite a variety of studies utilized to detect BM after BCS, it remains uncertain whether radionuclide is accurate in diagnosis for BM after BCS.^[12–27]^ We therefore will perform a rigorous systematic review to comprehensively compare the accuracy of radionuclide with x-ray, or computed tomography, or magnetic resonance.

## 2 Methods

### 2.1 Study registration

This study protocol was registered at CRD42020187646. It is reported according to the guidelines of the Preferred Reporting Items for Systematic Reviews and Meta-Analysis Protocol statement.^[28,29]^

### 2.2 Eligibility criteria for study selection

Studies will be eligible for inclusion if they are case-control studies (CCSs) on radionuclide in detection of BM after BCS; included patients who were diagnosed as breast cancer; compared BM with x-ray, computed tomography, magnetic resonance, regardless age, race, and severity of breast cancers; and reported outcomes of sensitivity, specificity, false negative rate, false positive rate, likelihood ratio, misdiagnosis rate, and diagnostic odds ratio.

We will exclude studies of animal studies, review, case report, case series, and non-clinical studies; and studies that did not focus on the radionuclide in detection of BM after BCS.

### 2.3 Strategy of literature retrievals

A systematic and comprehensive search of electronic databases (Cochrane Library, MEDLINE, EMBASE, Web of Science, CBM, and CNKI) will be performed until June 1, 2020. We will consider all eligible studies on the diagnosis of radionuclide in BM after BCS. The detailed search strategy is available for Cochrane Library in Table 1. We will create similar search strategies for other electronic databases. Besides the electronic databases, this study will also search grey literature, such as conference proceedings, websites of clinical trial registry, and reference lists of relevant studies.

**Table 1:**
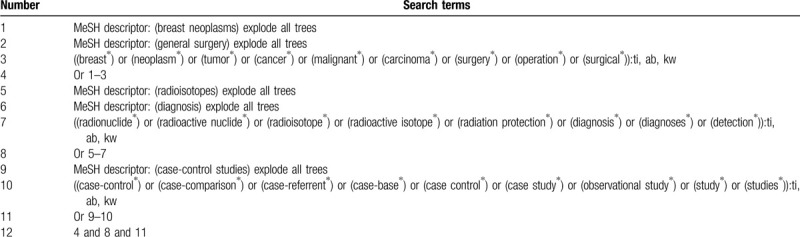
Search strategy of Cochrane Library.

### 2.4 Study selection

Two independent authors will examine titles/abstracts of all searched records, and will eliminate irrelevant studies. We will carefully check full-text of all potential studies to determine whether such studies meet all eligible criteria. The results of study selection will be shown in a flow chart. Any disagreement will be solved by consultation or discussion with a third author.

### 2.5 Data extraction and management

Two independent authors will extract data from included CCSs using a priori designed form. It comprises of study characteristics (e.g., study name, source date, and country), participant demographics and characteristics (e.g., age, diagnostic criteria, and sample size), details of index and reference tests, outcomes, results, and findings. Any doubt between 2 authors will be interpreted by a third author through discussion. If missing or insufficient data is identified, we will contact original authors to request it. If it is not successful, we will analyze available data.

### 2.6 Study quality assessment

We will appraise study quality using Quality Assessment of Diagnostic Accuracy Studies tool.^[30]^ Two authors will independently perform it. If there are conflicts regarding study quality evaluation, we will resolve them through discussion.

### 2.7 Measurements of treatment effect

Continuous data will be estimated by weighted mean difference or standardized mean difference and 95% confidence intervals (CIs); and dichotomous data will be estimated by risk ratio and 95% CIs.

### 2.8 Statistical analysis

Statistical analysis of this study will be completed using RevMan 5.3 software (London, UK). *I*^2^ test will be utilized to check statistical heterogeneity among included studies. *I*^2^ ≤ 50% denotes acceptable heterogeneity, and a fixed-effects model will be applied. If possible, we will also perform meta-analysis based on the sufficient similarity in study characteristics, patient demographic, and outcomes. *I*^2^ > 50% means significant heterogeneity, and a random-effects model will be placed. We will carry out subgroup analysis to explore sources of heterogeneity in accordance with different study information, patient characteristics, and study quality.

In addition, we will perform a sensitivity analysis to assess the impact of uncertain parameters on primary findings, and to check its stability and robustness by excluding low quality studies. We will also conduct funnel plot^[31]^ and Egger regression test^[32]^ to identify reporting bias if 10 eligible studies are available.

## 3 Discussion

BC is a rising major gynecological disease around the world.^[1,2]^ Although a range of studies reported the accuracy of radionuclide in diagnosis of BM in patients after BCS,^[12–27]^ there is still limited evidence-based medicine evidence to support this point. Thus, this systematic review will critically investigate the accuracy of radionuclide in diagnosis of BM after BCS. The results of this study may yield evidence to help judge whether or not radionuclide is accurate in diagnosis of BM after BCS. Its findings may benefit clinical practice and patients, as well as associated researchers.

## 4 Ethics and dissemination

This study does not need ethic approval, because we will not collect individual patient data. The results of this study will be published on a peer-reviewed journal.

## Author contributions

**Conceptualization:** Qi-xin Lian, Lian-jin Jin, Hao-jie Nie.

**Data curation:** Wei Zhao, Gang Li, Lian-jin Jin, Hao-jie Nie.

**Formal analysis:** Qi-xin Lian, Gang Li.

**Investigation:** Hao-jie Nie.

**Methodology:** Qi-xin Lian, Wei Zhao, Gang Li, Lian-jin Jin.

**Project administration:** Hao-jie Nie.

**Resources:** Qi-xin Lian, Wei Zhao, Gang Li, Lian-jin Jin.

**Software:** Qi-xin Lian, Wei Zhao, Lian-jin Jin.

**Supervision:** Hao-jie Nie.

**Validation:** Qi-xin Lian, Wei Zhao, Hao-jie Nie.

**Visualization:** Wei Zhao, Lian-jin Jin, Hao-jie Nie.

**Writing – original draft:** Qi-xin Lian, Gang Li, Lian-jin Jin, Hao-jie Nie.

**Writing – review & editing:** Qi-xin Lian, Wei Zhao, Gang Li, Hao-jie Nie.
